# Analyzing Gut Microbial Community in *Varroa destructor*-Infested Western Honeybee (*Apis mellifera*)

**DOI:** 10.4014/jmb.2306.06040

**Published:** 2023-07-24

**Authors:** Minji Kim, Woo Jae Kim, Soo-Je Park

**Affiliations:** 1 Department of Biology, Jeju National University, Jeju 63243, Republic of Korea; 2 Center for Life Science (HCLS), Harbin Institute of Technology, No.92 West Dazhi Street, Nangang District, Harbin City, Hei Longjiang Province, P.R. China

**Keywords:** Honeybee, *Apis mellifera*, *Varroa destructor*, microbiota, gut, larva

## Abstract

The western honeybee *Apis mellifera* L., a vital crop pollinator and producer of honey and royal jelly, faces numerous threats including diseases, chemicals, and mite infestations, causing widespread concern. While extensive research has explored the link between gut microbiota and their hosts. However, the impact of *Varroa destructor* infestation remains understudied. In this study, we employed massive parallel amplicon sequencing assays to examine the diversity and structure of gut microbial communities in adult bee groups, comparing healthy (NG) and *Varroa*-infested (VG) samples. Additionally, we analyzed *Varroa*-infested hives to assess the whole body of larvae. Our results indicated a notable prevalence of the genus *Bombella* in larvae and the genera *Gillamella*, unidentified *Lactobacillaceae*, and *Snodgrassella* in adult bees. However, no statistically significant difference was observed between NG and VG. Furthermore, our PICRUSt analysis demonstrated distinct KEGG classification patterns between larval and adult bee groups, with larvae displaying a higher abundance of genes involved in cofactor and vitamin production. Notably, despite the complex nature of the honeybee bacterial community, methanogens were found to be present in low abundance in the honeybee microbiota.

## Introduction

Next-generation sequencing has revolutionized the field of microbiome research, shedding light on the intricate relationships between microorganisms and their hosts in vertebrates (*e.g.*, humans) and plants [1, 2]. Studies investigating the microorganisms and microbial communities inhabiting the gastrointestinal tract of hosts have significantly advanced our knowledge of their ecologically functional roles in the gut environment. These microbiotas play critical roles in the survival of diverse host organisms, yet many aspects of their coevolution with hosts and their fundamental properties remain largely unexplored [3].

The western honeybee, *Apis mellifera* L, serves as a crucial pollinator for both natural ecosystems and agricultural production [4]. It plays a prominent role in pollinating over 90% of key commercial crops in the United States of America [5]; they pollinate 90% of blueberries and cherries, and the entire almond crop at bloom time (American Beekeeping Federation, http://www.abfnet.org). Furthermore, honeybees produce honey and beeswax, which are valuable commodities enriching their economic significance. However, the sustainability of honeybee colonies has been compromised by modern agricultural practices that involve the use of pesticides, agrochemicals, and exposure to pathogens, as well as environmental transformations like global warming [6-10]. One particular devastating threat to honeybee populations is the ectoparasitic mite *Varroa destructor*, which first appeared in *Apis cerana* and *Apis dorsata* and has since caused colony weakening and physical abnormalities such as deformed legs and wings [11]. Additionally, infectious diseases transmitted by viruses, microsporidia, or mites can impact honeybee health, activity, and population to varying degrees [12-15].

Recent research has highlighted the impact of gut dysbiosis induced by antibiotic and microplastic exposure on *A. mellifera* [16-19]. Additionally, antibiotic-resistant genes acquired by honeybees have been found to contribute to opportunistic human infections, despite the ban on antibiotic usage in the European Union [20]. Bacterial pathogens such as *Paenibacillus* larvae and *Melissococcus plutonius* also affect the microbial community of honeybees [21].

However, there are prospective applications for probiotics, such as *Lactobacilli* spp., which have been employed in honeybee colonies [22-24], with positive effects observed on honeybee activity, stress control, and queen brood production [23, 25-28].

The composition or transformation of the honeybee gut microbiota of different bee classes has been examined in numerous studies (*i.e.*, forage, nurse, or queen) [29-32], and has mostly focused on the entire body of honeybees or mites [33-37]. However, there is a lack of comprehensive investigations into the gut microbiota of adult honeybees infested with *V. destructor*.

This study aims to analyze the archaeal and bacterial community structures in larvae and adult honeybees from *Varroa*-infested hives. Additionally, we aim to infer differences in putative functional roles based on the microbial composition of larvae and adult bees. Our findings will contribute to a more comprehensive understanding of the interactions between gut microbiota and honeybees, as well as their functional roles within the honeybee gut environment.

## Materials and Methods

### Sample Collection

Beehives affected by *V. destructor* infestation were selected from beekeeping farms on Jeju Island, South Korea, towards the end of May 2021 (full blooming period begins to decline). These colonies were first discovered by an experienced beekeeper based on visual inspection of dead honeybees and clinical symptoms such as wing abnormalities. Prompt treatment of the infested hives with formic acid was conducted under the guidance of the Animal and Plant Quarantine Agency of South Korea. Owing to mite infestation leading to colony collapse, it was impossible to collect sufficient honeybees, larvae, and pupae. Fourteen adult bees were collected from *Varroa*-infested hives: unattached *Varroa* group (NG, *n* = 9) and attached *Varroa* group (VG, *n* = 5). In addition, we collected two larvae, but *V. destructor* presence could not be observed visually. Only living adults and larvae were selected for this study to minimize bias in the gut microbial community structure. The collected samples were immediately transferred to sterile falcon tubes, maintained at a low temperature (4°C) using an ice pack, and transported to the laboratory within 30 min for further processing, including genomic DNA extraction.

### DNA Extraction, Microbiota Quantification, and Amplicon Sequencing

Total genomic DNA (gDNA) was extracted from the isolated adult bee guts using a QIAamp PowerFecal Pro DNA Kit (Qiagen, Germany). As it is difficult to dissect the gastrointestinal tract from the larval body, whole-body larvae were used to extract gDNA. The entire gastrointestinal tract of each surface-sterilized adult honeybee was dissected under an anatomical microscope in a sterilized phosphate-buffered saline (PBS; pH 7.4). The quality and quantity of the extracted gDNA were estimated using a DS-11 Plus Spectrophotometer (DeNovix Inc., USA) and confirmed using agarose gel (1.5% w/v) electrophoresis. The gDNA samples were frozen at –20°C for subsequent experiments.

To quantify the bacterial and archaeal communities in each group, specific primer pairs for the bacterial and archaeal 16S rRNA genes were used as previously described [38, 39]. Quantitative real-time PCR (qPCR) experiments were performed using the CFX Connect Real-Time System (Bio-Rad Laboratories, USA) and built-in CFX Manager software (version 3.0; Bio-Rad Laboratories). To determine the abundance of the microbial community per ng of gDNA in each sample, standard curves were generated for each reaction using linearized gene standards (ranging from 0 to 10^8^ copies per run), as previously described [38]. Each sample was analyzed in triplicate by qPCR.

PCR was performed to obtain the amplicons for bacterial and archaeal 16S rRNA genes, according to previously described methods [39]. Briefly, the 20 μl system was used and prepared as follows: 10 μl of Solg 2x EF-Taq PCR Smart mix (Solgent, Korea), 1 μM primer set (final conc.), and ~5 ng of template gDNA. The procedures for thermal amplification were as follows: an initial denaturation step at 95 °C for 5 min; followed by 30 cycles of 95°C for 30 s, 55°C for 30s and 72°C for 40s, ended with a final extension step at 72°C for 7 min. The sequences of the primer sets targeted the V4–V5 hyper-variable region of the 16S rRNA gene for bacteria (515F, 5'-GTGCCAGCMGCCGCGGTAA-3' and 907R, 5'-CCGTCAATTCCTTTGAGTTT-3') and archaea (519F, 5'-CAGCCGCCGCGGTAA-3' and 915R, 5'-GTGCTCCCCCGCCAATTCCT-3'). The PCR-amplified products were visualized by 1.5% (w/v) agarose gel electrophoresis to confirm the amplified size. The amplicons were purified using the Monarch PCR & DNA Cleanup Kit (New England Biolabs, USA). High-throughput sequencing was performed with Novogene using an Illumina NovaSeq PE250 system (Illumina, USA) according to the manufacturer’s instructions.

### Data Analysis and Statistics

Sequencing data was analyzed following the standard operating procedure (SOP) provided by Mothur (version 1.46.1) [40, 41] (https://mothur.org/wiki/miseq_sop/). The barcode and primer sequences were trimmed to obtain raw reads. The trimmed paired-end reads were merged, and chimeric sequences were removed using the chimera.vsearch command. Non-microbial sequences (*e.g.*, chloroplast, mitochondria, and eukaryotes) were filtered out to improve analysis quality. The qualified-sequences were assigned to operational taxonomic units (OTUs) at 97% sequence similarity, with one representative sequence per OTU. Bacterial and archaeal sequences were aligned and classified using the silva.nr_v138.1 reference database provided by Mothur (https://mothur.org/wiki/silva_reference_files/). Alpha-diversity indices (Chao1 nonparametric richness, Shannon, inverse-Simpson, and Good's coverage) and beta-diversity indices [unweighted pair group method with arithmetic mean (UPGMA) clustering and principal coordinate analysis (PCoA)] were calculated using Mothur package. Analysis of similarity (ANOSIM) was performed to compare differences in microbial community structures. The taxonomic proportions for each sample or group (combined from the same experimental samples) of total sequences were calculated. Heatmaps and bar charts were generated using R packages (gplots and ggplot2, respectively). Linear discriminant analysis effect size (LEfSe) was utilized to identify taxonomic differences between the two groups with default parameters (logarithmic LDA score threshold > 2.0).

Putative functional profiles based on the microbial community were predicted using phylogenetic investigation of communities by the reconstruction of unobserved states (PICRISt2). The bacterial functional profiles were annotated using the Kyoto Encyclopedia of Genes and Genomes (KEGG) pathway.

### Availability of Data and Materials

The raw reads generated in this study have been deposited in the DDBJ/ENA/GenBank Sequence Read Archive (SRA) under the accession number PRJNA823814.

## Results

### General Features of Bacterial Diversity in Honeybee Gut Microbiota

A total of 2,531,114 raw reads were obtained from two larvae (designated as L) and 14 adult bees (VG, *n* = 5; NG, *n* = 9), with each sample sub-sampled to 20,000 reads to account for sample size variation [42]. After trimming and filtering, the number of reads used for sub-analysis ranged from 984 to 2689. The range for OTUs was estimated to 276-957 (Table 1). These results could be supported by the bacterial abundances in each sample ranged from 425 to 7.8 × 10^4^ copies were determined. This result could support the range of 276–957 OTUs (Table 1). Despite this variation, the average Good's coverage was 82.3%, suggesting a horizontal asymptote for microbial diversity (Table 1). Archaeal populations were not abundant in most samples (Table S1).

**Fig. 1:**
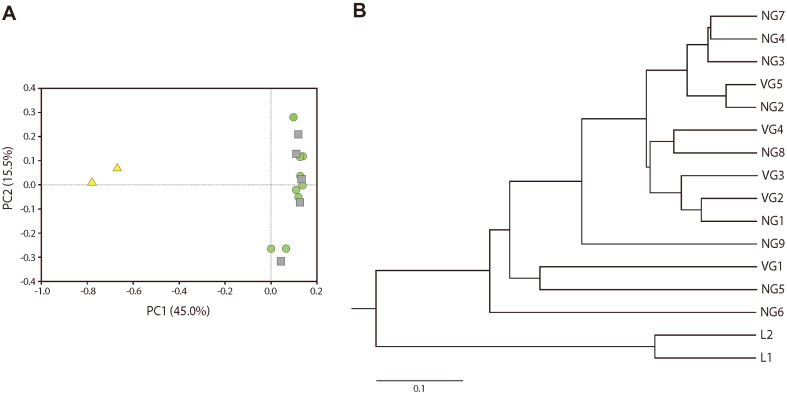
Relationships between the bacterial community profiles of the larva and adult bees. (**A**) Principal coordinates analysis (PCoA) plot representing the dissimilarity between samples based on Yue–Clayton metrics. The principal axes are shown with the percentage of variation explained in brackets. Each bee sample is denoted by larva (L, triangle, light yellow), non-*Varroa* group (NG, circle, light green), and *Varroa* group (VG, square, light gray). (**B**) Unweighted pair group method with Arithmetic Mean (UPGMA) clustering tree based on Yue–Clayton dissimilarity metrics.

The diversity indices of the larvae and adult bees were estimated based on qualified and subsampled reads (Table 1). The estimated OTUs (Chao1) indicated higher diversity in NG and VG compared to the L group (Fig. S1). However, no statistically significant differences were found between NG and VG (estimated Kruskal–Wallis test, *p* > 0.317). Microbiota of larvae showed significant differences compared to those of NG (ANOSIM, Global *R* = 0.985, *p* = 0.019) and VG (Global *R* = 1, *p* = 0.041), whereas NG and VG surprisingly displayed no significant differences in terms of gut microbiota (Global *R* = –0.071, *p* = 0.659). Taken together, despite the absence of statistically significant differences in diversity indices, the microbial community structures of larval and adult bee groups were clearly separated at the OTU level, as evident from the unweighted pair group method with arithmetic mean clustering (UPGMA) and principal coordinate analysis (PCoA) results (Fig. 1).

### Profiles of Honeybee Gut Bacterial Community

**Fig. 2:**
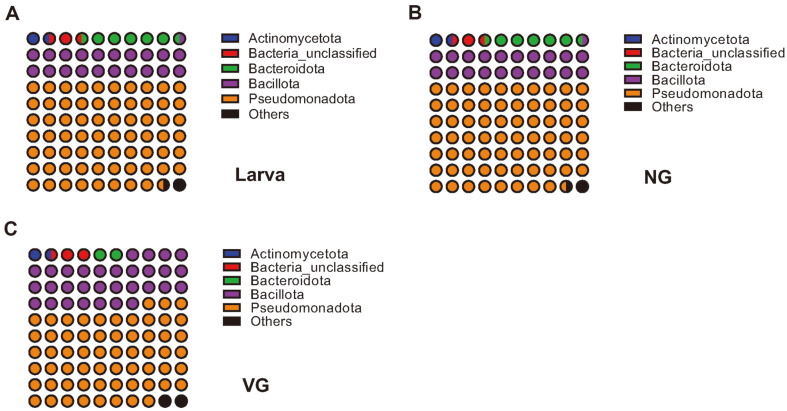
Relative abundances of the identified phyla in larva (A), non-*Varroa* group (NG) (B), and *Varroa* group (VG) (C) samples. Phyla abundances are represented by dot plots (10 × 10). Read sequences were assigned using the Mothur package and a reference database from the Silva database (version silva.nr_v138.1).

**Fig. 3:**
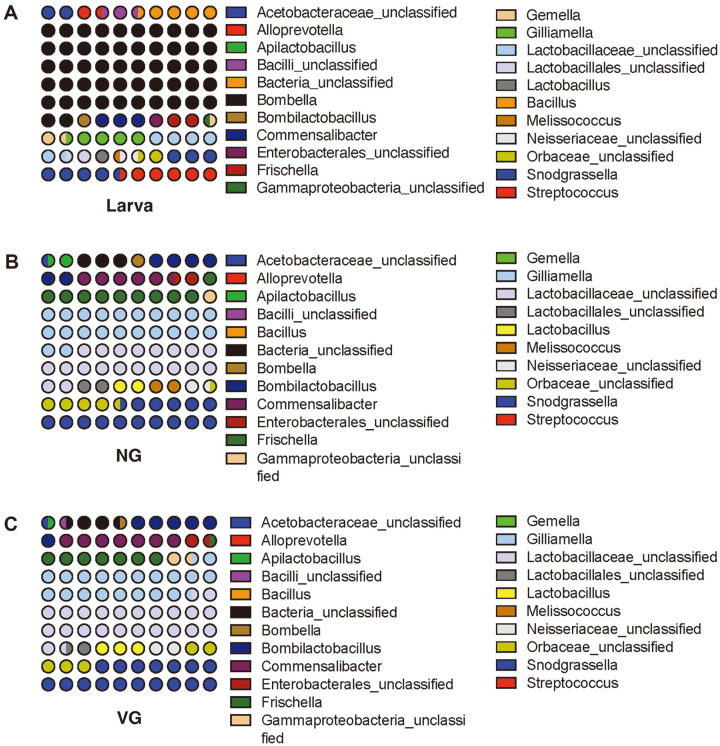
The abundances of the identified genera in the larva (A), non-*Varroa* group (NG) (B), and *Varroa* group (VG) (C) samples. Genera abundances represent by dot plot (10 × 10). The selected most relatively dominated genera (more than 1% of total read sequences in each group) are shown in stacked. Read sequences were assigned using Mothur package and a reference database of recently updated 16S rRNA gene obtained from the Silva database (version silva.nr_v138.1).

Although alpha-diversity analysis indicated no discernible differences (*i.e.*, diversity indices, Table 1), there were considerable differences in microbial communities among developmental stages (larvae and adults) (Figs. 2 and 3) [43].

The analyzed sequence reads were classified into 40 phyla. We focused on the relative abundance of the five most abundant phyla (>1% of total reads) in each group (L, NG, and VG) (Figs. 2 and S2a). Pseudomonadota (59.6%–68.7%) and Bacillota (20.4%–30.7%) were identified as the most abundant phyla (> 20% of total reads) in the three groups (L, NG, and VG), followed by Bacteroidota, an unidentified group, and Actinomycetota (>1% of total reads). Among minor phyla (< 1% grouped into other), Campylobacterota was more abundant in adult bee groups (NG and VG) than in larvae. In contrast, Gemmatimonadota, Myxococcota, Synergistota, Verrucomicrobiota, and Bdellovibrionota were more abundant in NG than in L and VG. At the family level, Orbaceae, Lactobacillaceae, and Neisseriaceae exhibited higher abundance compared to the L group (Fig. S2b). However, Acetobacteraceae (47.53%) showed higher abundance in the L group than in NG and VG (5.22% and 6.78%, respectively). Melioribacteraceae and Streptococcaceae were slightly more abundant in the L group (1.23% and 5.08%, respectively) compared to NG and VG (less than 1%).

The analyzed sequence reads were classified into 727 genera (Figs. 3 and S2c). The majority of the reads belonged to unclassified taxa at higher taxonomic ranks (family to class) . We selected 22 genera from each group (threshold > 1% of total reads) for further analysis. Nine significant taxa (>4% of each group), comprised *Bombella*, *Bombilactobacillus*, *Commensalibacter*, *Frischella*, *Gilliamella*, unclassified Latobacillaceae, unclassified Orbaceae, *Snodgrassella*, and *Streptococcus*, were identified. The genus *Bombella* had the highest relative abundance in the L group (43.7% of total bacterial abundance), which decreased in adult bees (less than 0.6%). Several taxa in the L group showed higher relative abundance compared to the adult bee group, including *Streptococcus*, *Alloprevotella*, *Bacillus*, *Gemella*, and other high-taxonomic groups (*e.g.*, Acetobacteraceae and Bacilli) (Fig. S3). In NG and VG, *Gilliamella*, unidentified Lactobacillaceae, and *Snodgrassella* (12.8%–19.7%) were identified as the dominant microbes. Additionally, the unclassified Enterobacterales, unclassified Gammaproteobacteria, *Lactobacillus*, and unclassified Neisseriaceae showed an increased abundance in the NG and VG groups compared to in the L group. Except for the genera *Apilacetobacillus*, *Melissococcus*, and *Lactobacillus*, the relative abundances of the other genera were comparable between NG and VG (Fig. 3).

**Fig. 4:**
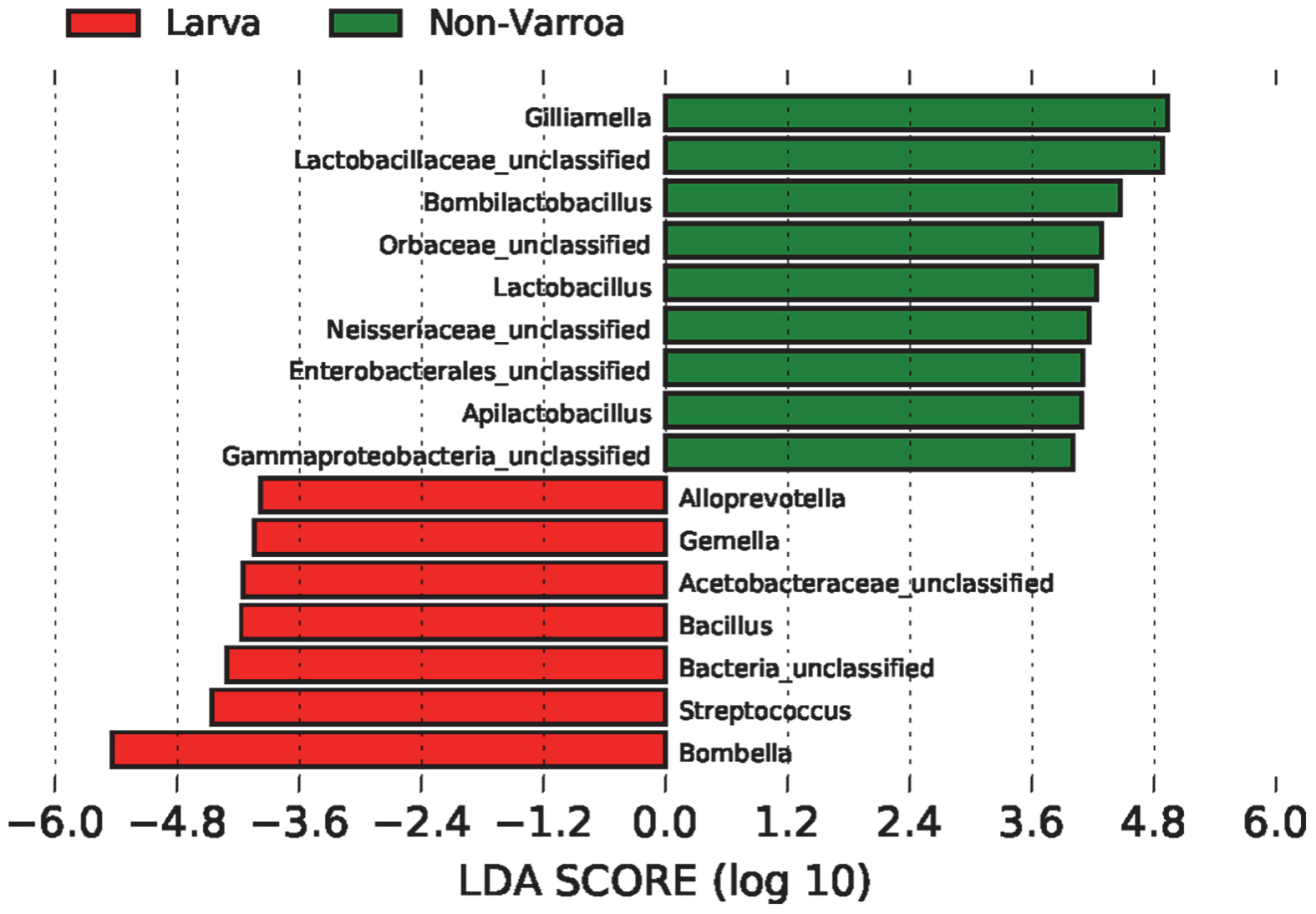
Linear discriminant analysis effect size (LEfSe) analysis results presented as bar charts showing the linear discriminant analysis (LDA) scores. LDA scores indicate significant bacterial differences between larva and NG at the selected genera. The groups were statistically significant compared to each other (LDA > 2.0 and *p* < 0.05).

LEfSe analysis was conducted to identify distinctive taxa at the genus level between NG and VG (Fig. 4); however, no significant differences were found. Only two genera, *Melissococcus* and *Apilbactobacillus*, were identified as distinguished taxa in NG (Fig. S4a), and they were found to be exclusively present in NG (Figs. 3 and S2). To further assess the precise variations in microorganisms between the L, NG (Fig. 4), and VG (Fig. S4b) groups, we quantified the relative enrichments of genera. Seven genera, namely *Bombella* (5.44 LDA score, *p* = 0.0001), *Streptococcus* (4.47 and 0.0001), unclassified bacterial group (4.44 and 0.0001), unclassified Acetobacteraceae (4.35 and 0.0001), *Bacillus* (4.31 and 0.0001), *Gemella* (4.11 and 0.008), and *Alloprevotella* (4.11 and 0.0001), showed significant enrichment in the L group. *Gilliamella*, unclassified Lactobacillaceae, unclassified Orbaceae, unclassified Neisseriaceae, *Frischella*, *Lactobacillus*, unclassified gammaproteobacteria, *Bombilactobacillus*, and unclassified Enterobacterales were indicated as dominant genera in the NG with an LDA score of 4.43–4.93 and *p* < 0.04 (Fig. 4). Interestingly, the two genera *Commensalibacter* and *Snodgrassella* were predominant taxa in VG, similar to NG (Fig. 3). However, LEfSe analysis did not reveal any significant taxa in VG (Fig. 4b).

### Honeybee Gut Archaeal Community Profiles

In this study, we aimed to determine the archaeal community profiles of honeybees, including larvae. We found a relatively limited archaeal community in the 10 samples, consisting of one larva and nine adult bees (NG, *n* = 5; VG, *n* = 4). The genus *Methanomassiliicoccus*, belonging to the phylum Thermoplasmatota, was the dominant archaeon in larvae. In NG, Methanimicrococcus (80.5% of total reads) and *Candidatus* Methanoplasma (17.3%) were the most common archaeal taxa, belonging to the phylum Halobacteria and Thermoplasmatota, respectively. An unidentified archaeal group with an abundance of 2.0% was also identified in NG. Interestingly, when comparing NG and VG, the dominant genus *Methanomassiliicoccus* was found to comprise 98.7% of the total reads in VG.

### Predicted Functional Profiles from Bacterial Communities

**Fig. 5:**
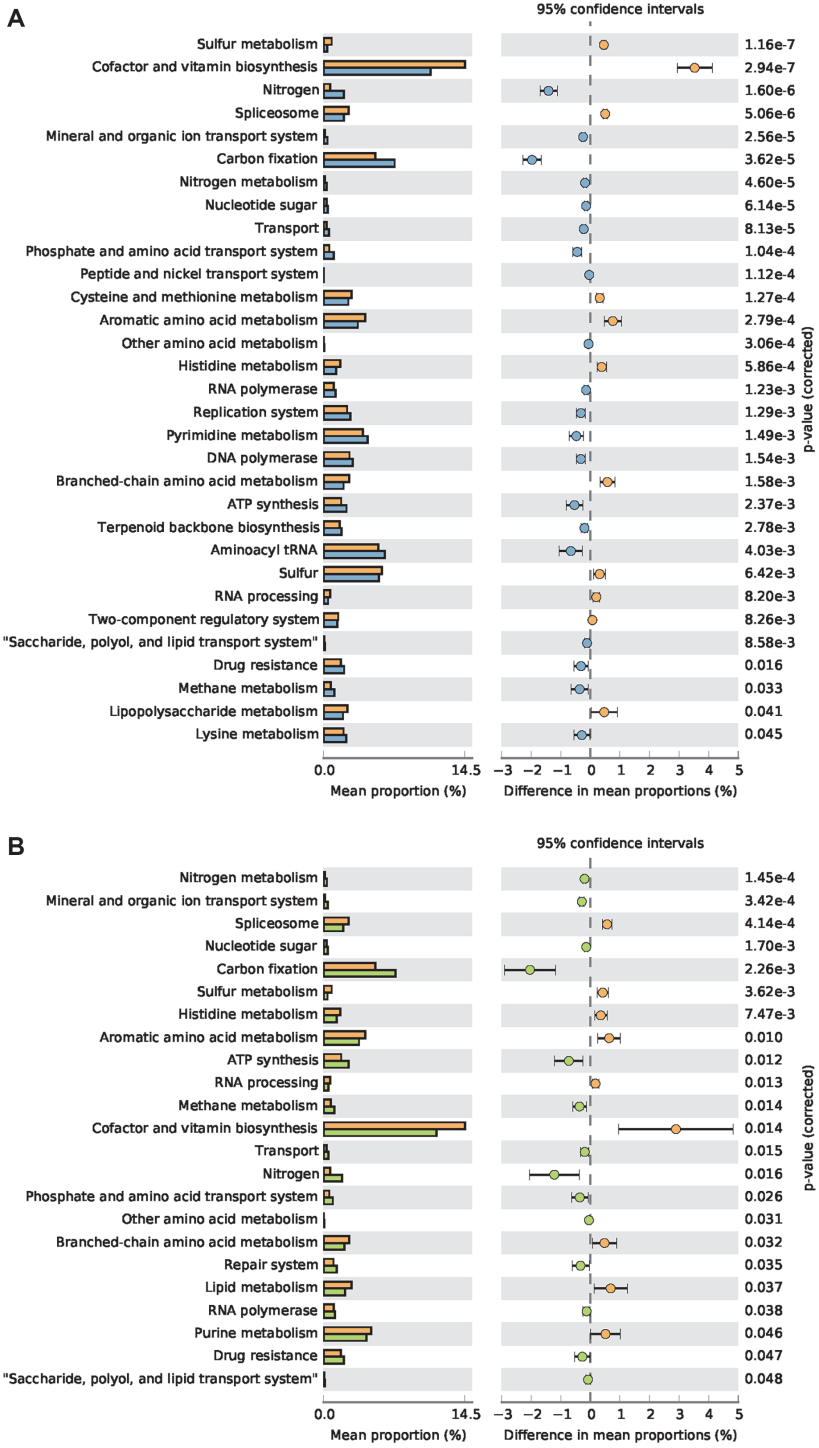
PICRUSt analysis. The chart for the predicted functional characterization at KEGG level 3 significant difference (*p* < 0.05) between larva and non-*Varroa* group (NG) (**A**), and *Varroa* group (VG) (**B**), presented using STAMP software. Larva, orange; NG, blue, *Varroa* group (VG, green).

Inferring functional roles based on microbial community organization, as determined by the 16S rRNA gene, can be challenging. In this study, PICRUSt analysis and KEGG pathway information were utilized to infer putative functional profiles for inter-group comparisons. The results of the KEGG functional classes (levels 1 and 2) revealed substantial differences among the L, NG, and VG groups in terms of functional categories (Fig. 5). However, no significant variations were observed between NG and VG in the PICRUSt analysis, consistent with the findings of alpha diversity and LEfSe (Table 1 and Fig. 4).

In comparison to NG and VG, the L group exhibited significant effect validity in eight functional categories: lipid metabolism, spliceosome, sulfur metabolism, cofactor and vitamin biosynthesis, RNA processing, histidine metabolism, aromatic amino acid metabolism, and branched-chain amino acid metabolism (effect size ranging from 0.37 to 0.82). Notably, the biotin synthesis gene clusters in the L group showed a distinct enrichment compared to adult bees (Fig. 5). The validity of carbon fixation, methane metabolism, mineral and organic ion transport systems, nitrogen metabolism, glycosaminoglycan metabolism, nitrogen, nucleotide sugar, repair system, transport, peptide and nickel transport systems, and phosphate and amino acid transport systems was relatively low in the L group.

## Discussion

The gut microbiota plays a significant role in the overall health and functioning of organisms, including plants. Recent studies have revealed that the number of microbial cells, particularly bacteria, is comparable to that of human cells, challenging the previous estimate of a 10-fold difference [44]. Despite the relatively small ratio, the gut microbiota can still make a substantial impact on host health and disease. In invertebrates, such as bees, the complex microbial community has been found to be closely linked, but the specific physiological roles of the bee microbiota in health and development stages are still not well understood [45, 46]. Advances in next-generation sequencing and culture-dependent techniques have considerably enriched our understanding of the relationship between bees and associated bacteria, and the role of the gut microbiota in healthy adult worker honeybees has been recognized.

The objective of this study was to investigate differences in the gut microbiota between *Varroa*-infested (attached) and uninfested bees. The archaeal and bacterial populations of the samples were analyzed using Illumina NovaSeq technology and the 16S rRNA gene amplicon. As previously reported, Bacteroidota (formerly termed Bacteroidetes) and Bacillota (formerly termed Firmicutes) are the predominant taxa in the vertebrate gut microbiota [39]. However, our findings revealed that the larval and adult groups were dominated by Pseudomonadota (formerly *Proteobacteria*) and Bacillota phyla, consistent with several other studies on honeybees [43, 47-50]. We identified the primary phyla and genera in each experimental group and found significant differences in bacterial communities between the L and adult bee groups (NG and VG). However, biostatistical analysis indicated no difference in alpha diversity and gut bacterial community structure between NG and VG, which contradicted the qPCR results that detected specific bacteria in adult bees from *Varroa*-infested colonies [37]. In addition, previous studies have reported the transmission of certain microorganisms between honeybees and *Varroa* mites [36]. However, it is important to note that our sampling focused only on the gut flora, whereas the two previously cited studies evaluated the total body of bees.

Notably, Bacteroidota exhibited a higher relative abundance (2.16%–5.75%) than Actinomycetota (1.28%–1.62%) in all samples (Figs. 2 and S2), consistent with the results of previous studies [48, 51]. In particular, the abundance of Bacteroidota in the L group was higher than that in adult bees. However, the Bacteroidota abundance observed in adult bees was higher than that reported in other studies [43, 49, 50]. These differences may be attributed to experimental differences, such as the targeted region of the 16S rRNA gene (*e.g.*, V1–V3 or V3–V4) [52].

Bacteroidota is considered a prominent taxon in both mammalian and insect gut microbiota [53-55]. It possesses the ability to degrade soluble polysaccharides and utilize them through loci-like systems [54]. The extracellular enzymes produced by Bacteroidota bacteria can contribute to vitamin synthesis within the host through intra- or intercellular reaction chains [56]. However, owing to its relatively low abundance, the role of Bacteroidota in honeybees is less understood compared to other taxa, such as Bacillota [46].

Consistent with previous studies, distinct genera were identified in both the larval and adult bee groups. Specifically, the L group was dominated by reads related to the genus *Bombella*. The genus *Bombella* was first proposed by Li *et al*. [57] as a member of Alphaproteobacteria, with *Bombella intestini* described as the type species isolated from bumble bee crops. The genus *Bombella* contains only four validly named species [57-59]. Interestingly, these *Bombella* spp. have only been isolated from honeybee-associated environments, such as honeycombs and the gut. In addition, all members of the genus *Bombella* share unique characteristics related to acetic acid production. The Acetobacteraceae Alpha 2.2 bacteria of the genus *Bombella* have been speculated to play a functional role in the fitness of young larvae [60]. Recent genomic studies have further highlighted the critical role of *Bombella* in host interactions [61, 62]. Among several other genera and high-taxonomic groups, *Allopreovotella* was identified as a minor group within the L group (*e.g.*, *Acetobacteraceae* and Bacilli) (Figs. 3 and S2c). To the best of our knowledge, only one previous study has identified the genus *Alloprevotella* in adult bees [43]. *Alloprevotella*, reclassified from *Prevotella* [63] is capable of producing acetate and succinate (*i.e.*, short-chain fatty acids, SCFAs) through glucose fermentation. SCFAs are important metabolites produced by gut bacterial fermentation of saccharides (*e.g.*, starch or fiber) and contribute to various physiological effects on host health, including immunity, behavior, and neurological disorders [64, 65]. The status of the *Prevotella* spp. in the host remains controversial among various investigations [66]. The beneficial commensal species *Allopreovotella* spp. cannot be easily dismissed, as it is essential for larval health through polysaccharide breakdown and SCFA production.

Comparing the gut microbiota of the L group to the adult bee groups, a distinct microbial community comprising four different classes (*e.g.*, Bacilli, Alpha-, Beta- and Gamma-proteobacteria) was observed (Fig. 2). This difference could be attributed to the four distinct developmental stages (egg, larva, pupa, and adult) and the distinct feeding systems of nursing bees for larvae (pollen and honey) [46, 67]. Several genera, including *Bombilactobacillus*, *Commensalibacter*, *Frischella*, *Gilliamella*, *Lactobacillus*, and *Snodgrassella*, have been extensively studied and their roles in honeybees are well characterized [29, 68-70]. Additionally, the genus *Bifidobacterium*, a core bacterial clade, may play a role in degrading organic or aromatic compounds derived from pollen [71]. These pollen-derived compounds can be cross-absorbed by other gut bacteria, contributing to bee development [72]. Although limited studies have linked gut microbiota and *Varroa* infection, some studies have reported the detection of *Bifidobacterium* in the gut microbiota, with its abundance positively correlated with *Varroa* infection [35, 37]. Unexpectedly, the relative abundance of *Bifidobacterium* (phylum Actinomycetota) was less than 0.5% of the total microbiota in both the L and adult bee groups in our study. However, the relative abundance of unclassified *Bifidobacteriaceae* was similar to that of *Bifidobacterium*, suggesting that more unclassified species belonged to the family *Bifidobacteriaceae* in the honeybee gut. Lactic acid bacteria, including *Bifidobacterium*, contribute to host defense against pathogens by lowering the gut pH through the production of organic acids and antimicrobial substances such as antimicrobial peptides (AMPs) [73]. Therefore, further investigation is needed to clarify their potential activities and their comprehensive functional roles in pathogen defense in honeybee gut microbiota.

In this study, we also analyzed and identified the archaeal community in the entire honeybee gut for the first time. Unexpectedly, the archaeal diversity and community structure were extremely limited. Only a few bee samples harbored methanogens, despite the anoxic conditions with a partial oxygen pressure close to zero in the honeybee gut [74]. This could be due to the positive redox potential (215–370 mV) in the honeybee gut, as methanogenesis is more commonly observed under anaerobic conditions with a negative redox potential (–200 mV) [75, 76]. Insects such as beetles, cockroaches, termites, and millipedes are known to possess methanogens or other archaeal groups in their hindguts [53, 77].

This study has certain limitations. Firstly, the analysis of the gut microbiota was conducted a few days after formic acid treatment for *Varroa* infection. Examining the time course of *Varroa* infection in the honeybee gut would provide more detailed information about gut microbiota transformation and dysbiosis. Secondly, the sample size for each group was relatively small, despite consistent findings in previous studies.

In summary, this study provides valuable insights into the developmental stages of honeybees based on the organization of the gut bacterial community. The larval and adult bee groups exhibit distinct bacterial compositions and distributions. The predicted functional profiles of these groups also differ based on their bacterial communities. However, the functional characteristics were comparable between non-*Varroa* and *Varroa* groups. The observed bacterial and archaeal community structures in honeybees are likely essential factors contributing to their overall health.

### Outlook

Characterizing the microbial composition and isolating key microorganisms from the gut microbiota is a challenging task thus far. Many microorganisms remain uncultured, and the specific impacts of individual microorganisms cannot be fully examined using molecular techniques such as next-generation sequencing. In addition, our understanding of the relationship between humans and the gut microbiota, as well as its physiological involvement in the host gut, is still limited. In comparison, invertebrate organisms, including insects, harbor relatively simple gut microbial communities [53]. Model organisms like the fly *Drosophila* [78] and honeybees, in particular, have been employed to study social behavior, brain disorders, aging, and development [79-82]. Moving forward, it is crucial to continue exploring and understanding the gut microbiota. This includes efforts to culture and identify previously uncultured microorganisms and investigate their specific roles. Additionally, utilizing honeybees as a model organism can provide valuable insights into the functional roles of the gut microbiota and its impact on host health. By advancing our knowledge of the gut microbiota in honeybees and other organisms, we can gain a deeper understanding of the intricate relationship between microorganisms and their hosts, ultimately leading to important discoveries and potential applications in various fields [46, 70].

## Supplemental Materials

Supplementary data for this paper are available on-line only at http://jmb.or.kr.



## Figures and Tables

**Table 1:** Overview of estimates of read sequence diversity and phylotype coverage of NovaSeq data generated from larva and adult bee samples.

Group^a^	Analyzed reads	OTU^b^	Chao1	Shannon	Simpson^c^	Good’s coverage	Bacterial abundance^d^
L1	1049	306	889.03	4.65	14.88	0.80	425.70±4.32
L2	984	295	893.78	4.54	12.84	0.79	1397.20±3.55
NG1	1479	343	1096.38	4.28	12.62	0.83	5170.29±52.52
NG2	1516	336	1372.03	4.10	10.76	0.83	29,875.68±151.76
NG3	1829	370	1425.68	4.09	11.30	0.85	45,940.38±700.02
NG4	1750	333	1134.24	4.06	12.69	0.86	21,011.64±106.73
NG5	2689	957	2046.66	6.20	76.81	0.78	34,351.13±959.45
NG6	1494	407	1304.06	4.74	19.81	0.80	38,147.12±96.89
NG7	1663	337	1294.03	4.03	12.36	0.84	42,258.16±536.61
NG8	1596	438	1589.63	4.66	16.16	0.79	63,283.78±3372.10
NG9	1396	276	979.64	3.76	9.32	0.85	78,337.94±3181.66
VG1	1078	316	951.63	4.72	20.48	0.79	28,860.83±1318.50
VG2	1620	350	1476.44	4.07	11.61	0.83	28,369.44±1008.31
VG3	1567	419	1634.24	4.52	13.50	0.80	60,108.58±1526.31
VG4	1723	301	1177.72	3.63	7.89	0.87	45,362.68±1727.35
VG5	1681	332	1131.03	4.10	11.09	0.86	9437.09±95.87

The diversity indices and richness estimators were calculated using Mothur software. Diversity was estimated using operational taxonomic units (OTUs) and was defined as groups with ≥97% sequence similarity.

^a^L, NG, and VG denote the larval, non-*Varroa*, and *Varroa* groups, respectively.

^b^The OTUs were determined based on 97% of 16S rRNA gene similarity.

^c^Inverse-Simpson (see the materials and methods)

^d^16S rRNA gene copies per ng (gDNA) estimated by qPCR, and data are means ± standard deviation from triplicate reactions (see Materials and Methods).
