# Supplementary material for: Analyzing Gut Microbial Community in Varroa destructor-Infested Western Honeybee (Apis mellifera)
Source: J Microbiol Biotechnol. 2023 Jul 24;33(11):1495–505. doi: 10.4014/jmb.2306.06040 (PMC10699279; doi:10.4014/jmb.2306.06040)

Supplementary Table and figure legends

Table S1. Relative abundance of archaeal groups identified in this study.

	L (n=1)	NG (n=5)	VG (n=4)
Archaea_unclassified	0.0%	2.0%	0.0%
Halobacterota	0.0%	80.5%	1.3%
Thermoplasmatota	100.0%	17.3%	98.7%

*Total number of analyzed reads classified into archaea was less than 1,000 in each group

Figure S1. Alpha-diversity measures, including Chao1 (A), Shannon richness (B), and inverse Simpson diversity (C) indices, plotted for larva (L, light yellow), NG (light green), and VG (light gray) groups. These values were calculated with the Yue–Clayton dissimilarity metric based on the proportions of OTUs in different samples. The plots are based on the data shown in Table 1. The horizontal line inside the box indicates the median value. The whiskers represent the lowest and highest values within 1.5 times the interquartile range (IQR) from the 25th and 75th percentiles. Outliers, as well as individual sample values, are shown as dots.

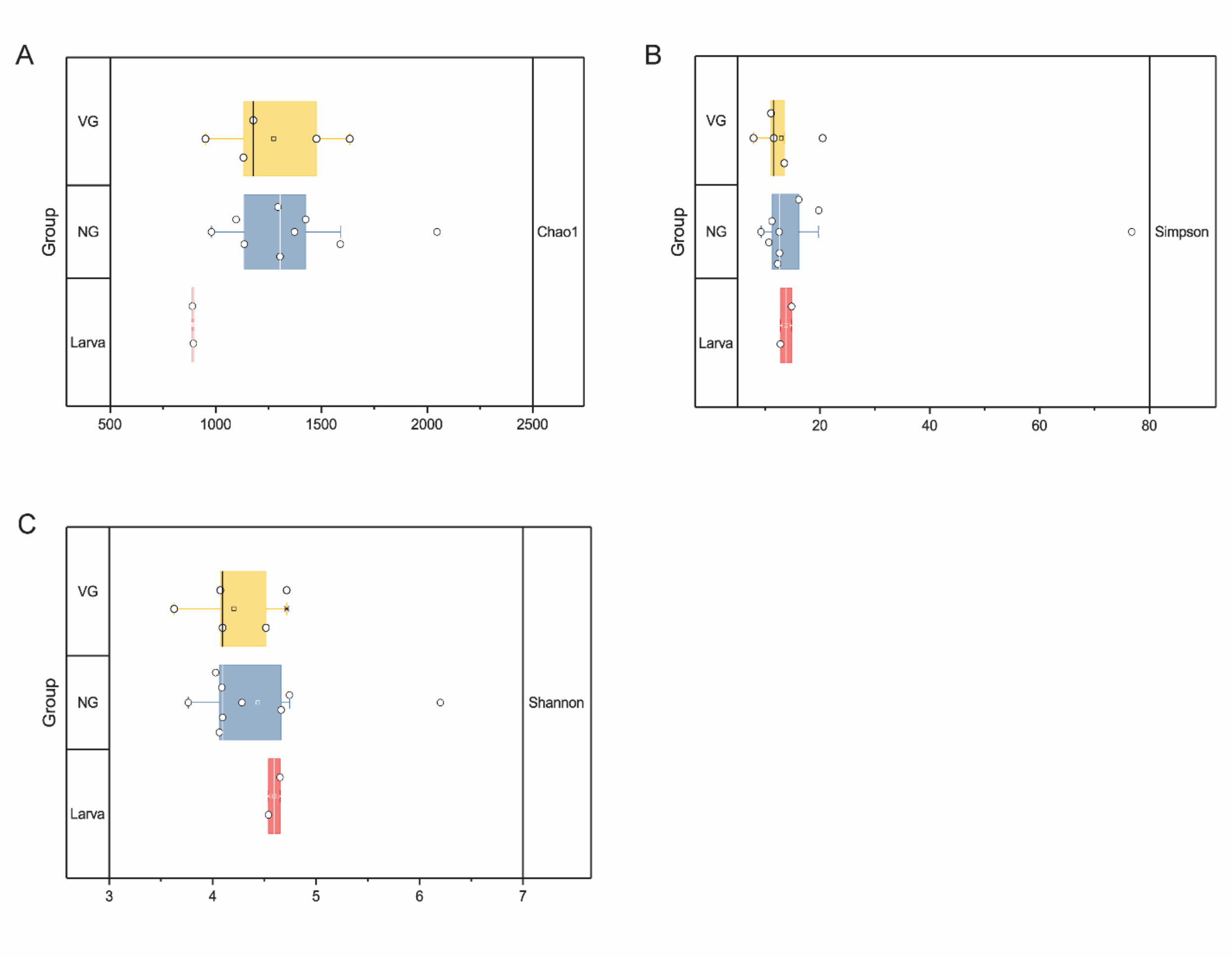

Figure S2. Stacked bar chart representing the relative abundances evaluated at the phylum (A), family (B), and genus (C) taxonomic level in the bacterial communities of the larva (L), non-*Varroa* (NG), and *Varroa* group (VG).

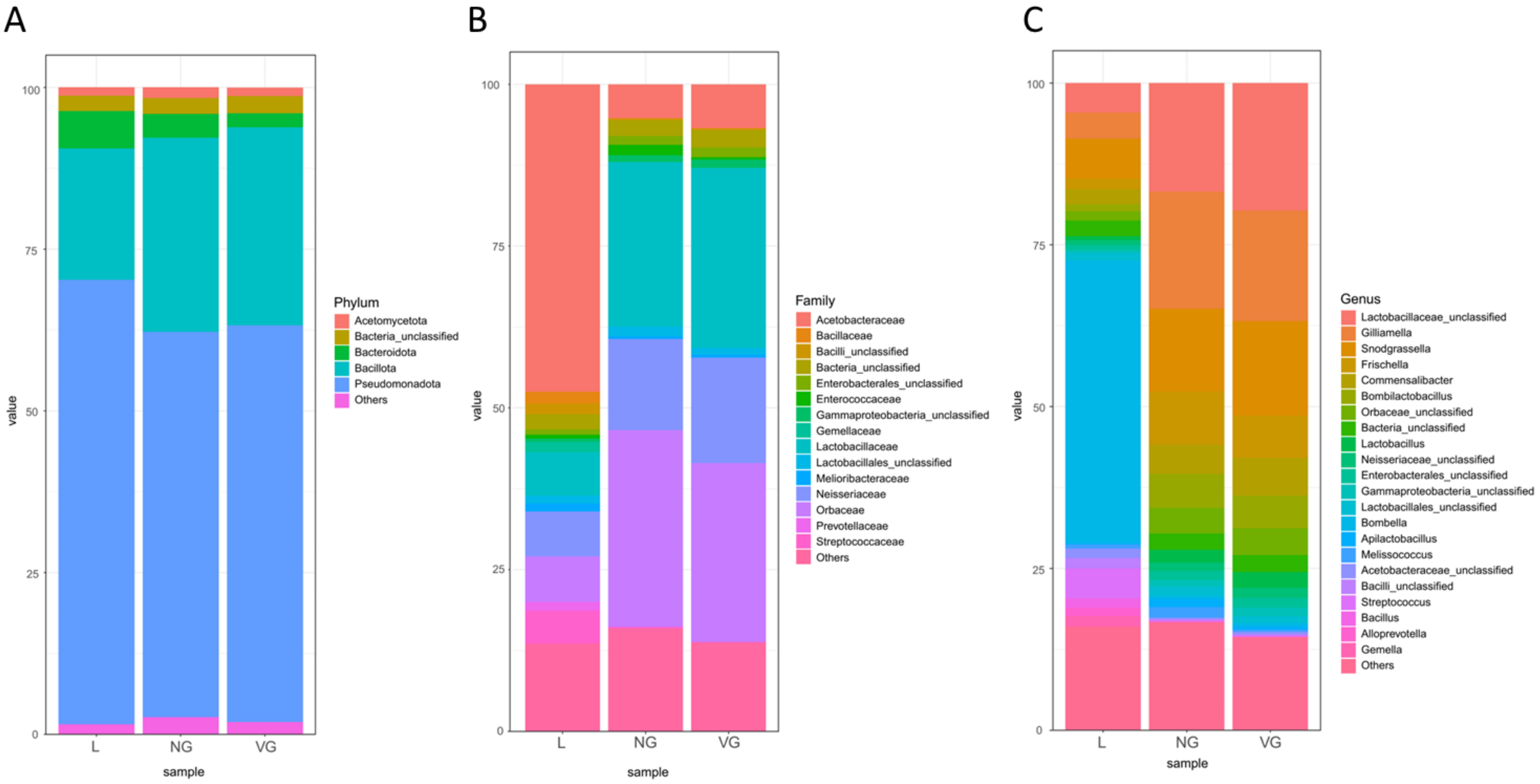

Figure S3. Heatmap showing the selected most relatively dominant genera (more than 1% of total read sequences) in each group. The heatmap was generated using the gplot package.

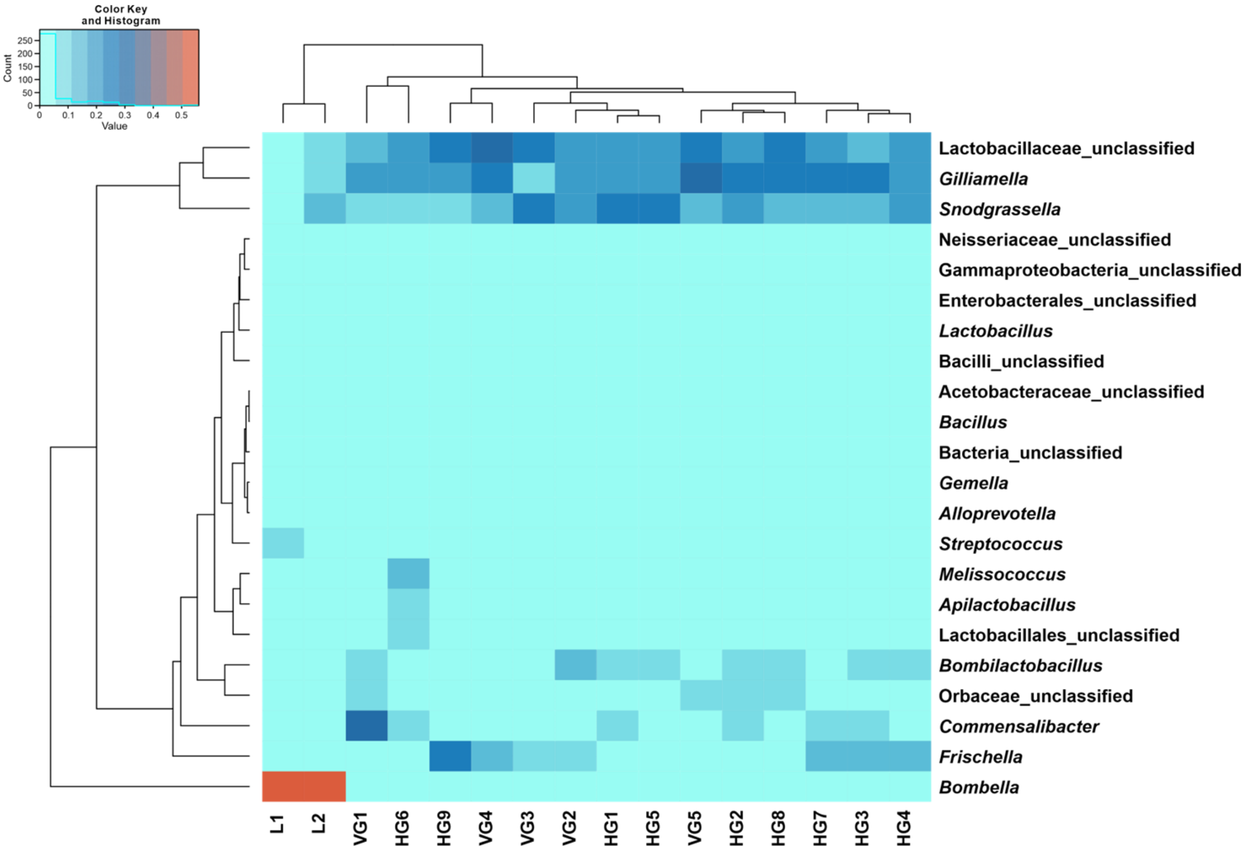

Figure S4. LEfSe analysis results presented as bar charts showing the LDA scores. LDA scores indicate significant bacterial differences between NG and VG (A), and between L and VG (B) at the selected genera. The groups were statistically significant compared to each other (LDA > 2.0 and $p < 0.05$).

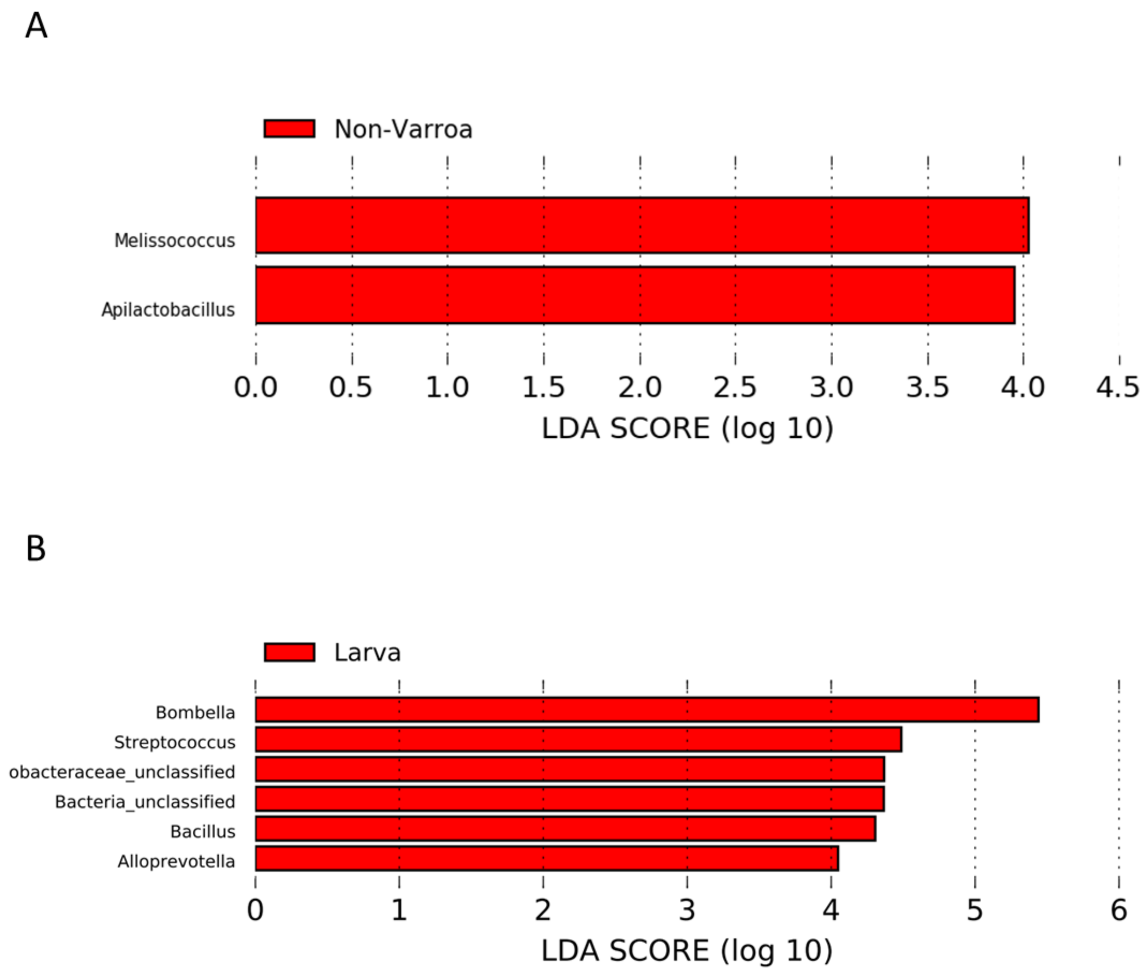

Supplement: Supplementary file 1 [file jmb-33-11-1495-supple.pdf]
